# Effect of seed's geographical origin on cactus oil physico-chemical characteristics, oxidative stability, and antioxidant activity

**DOI:** 10.1016/j.fochx.2024.101445

**Published:** 2024-05-07

**Authors:** Issmail Nounah, Said El Harkaoui, Ahmed Hajib, Said Gharby, Hicham Harhar, Abdelhakim Bouyahya, Giovanni Caprioli, Filippo Maggi, Bertrand Matthäus, Zoubida Charrouf

**Affiliations:** a Department of Chemistry, Faculty of Sciences, Mohammed V University in Rabat, BP 1014 Rabat, Morocco; b Max Rubner-Institut, Federal Research Insitute for Nutrition and Food, Department of Safety and Quality of Cereals, Schützenberg 12, D-32756 Detmold, Germany; c Higher School of Education and Training (ESEF), Université Ibn Zohr, Agadir, Morocco; d Biotechnology Analytical Sciences and Quality Control Team, Polydisciplinary Faculty of Taroudant, Université Ibn Zohr, Morocco; e Laboratory of Materials, Nanotechnology and Environment LMNE, Faculty of Sciences, Mohammed V University in Rabat, BP 1014 Rabat, Morocco; f Laboratory of Human Pathologies and Biology, Faculty of Sciences, Mohammed V University in Rabat, Morocco; g Chemistry Interdisciplinary Project (ChIP), School of Pharmacy, University of Camerino, Via Madonna delle Carceri, 62032 Camerino, Italy

**Keywords:** Chemical composition, Geographical origin, *Opuntia ficus-indica*, Phenolic content, Tocopherols

## Abstract

The aim of this study was the valorisation of cactus (or prickly pear, *Opuntia ficus-indica*) seeds growing in six different regions of Morocco. Moisture, proteins, lipids profile, total polyphenols content, oxidative stability, and antioxidant activity were investigated. The Folin-Ciocalteu test highlighted the abundant presence of phenolic compounds (165 to 225 mg EAG/100 g of extract) and a significant antioxidant capacity against DPPH free radicals. The seeds contained protein (7–9.25%) and lipids (2.7–5%). Cactus oil quality indices such as acidity and peroxide value were below 1.2% and 10 mEq.O_2_/kg, respectively. GC analysis revealed that linoleic and oleic acid percentages ranged from 57.1 to 63.8%, and 13.5 to 18.7%, respectively. Cactus seed oil was rich in tocopherols (500–680 mg/kg) and phytosterols (8000–11,100 mg/kg) with a predominance of γ-tocopherols and β-sitosterol. Triacylglycerols, fatty acids and sterols composition showed small variation depending on the geographical origin, while the individual tocopherol profile was significantly influenced.

## Highlights

• Proximate analyses have been performed on cactus from six different Morocco regions.
• Cactus oil quality indices such as acidity and peroxide value were optimal.
• Cactus seed oil was rich in tocopherols (500–680 mg/kg) especially γ-tocopherols.
• Cactus seed oil was rich in phytosterols (8000–11,100 mg/kg) especially β-sitosterol.
• Individual tocopherol profile was influenced by geographical origin.

## Graphical abstract

**Figure:**
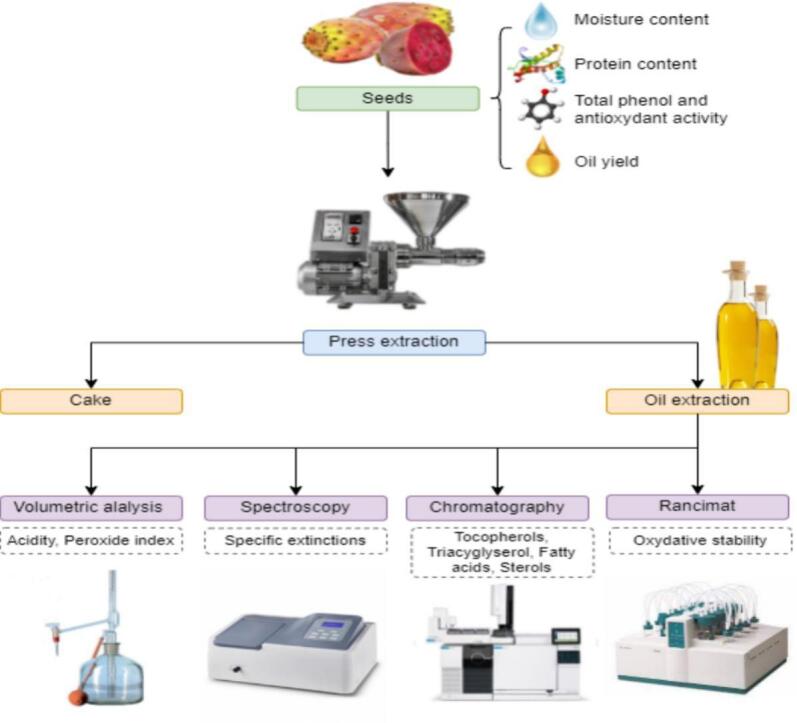

## 1 Introduction

Cactus (*Opuntia ficus-indica* (L.) Mill.), is a shrub of the Cactaceae family, mainly growing in arid and semi-arid areas (America, the Mediterranean, Africa, the Middle East, Australia and India) (Barbera, Inglese, Pimienta-Barrios, & Arias-Jiménez, 1995). This species is of great agronomic importance, both for edible fruit and cladodes, which can be used as fodder or vegetable (Mulas & Mulas, 2004)**.** Furthermore, cactus flowers are a source of nutrients highly valued by bees, hence the possibility of developing beekeeping (Arba, 2009). The seeds are rich in minerals, predominating phosphorus (152 mg/100 g) and potassium (163 mg/100 g). The seeds also contain important amounts of magnesium (74.8 mg/100 g), sodium (67.6 mg/100 g), and calcium (16.2 mg/100 g) (Chavez-Santoscoy, Gutierrez-Uribe, & Serna-Saldívar, 2009; El Kossori, Villaume, El Boustani, Sauvaire, & Méjean, 1998). In addition, they are rich in phenolic compounds (268.4 mg/100 g) and also contain 6.0% of protein and 5.5% of oil (Tlili, Sakouhi, Elfalleh, Triki, & Khaldi, 2011)**.** Cactus seed oil belongs to the oleic-linoleic oils group, which makes it particularly interesting in cosmetic, nutritional and pharmacological fields (Chbani, El Harkaoui, Willenberg, & Matthäus, 2023; Coşkuner & Tekin, 2003; Ramadan & Mörsel, 2003). The oil is also rich in tocopherols and phytosterols known for a wide spectrum of pharmacological activities (Chbani et al., 2023)**.** Alqurashi, Al Masoudi, Hamdi, and Abu Zaid (2022) reported the importance of cactus seed oil as antioxidant, antibacterial, antifungal, and anticancer agent (Alqurashi et al., 2022)**.**

Recently, many women's cooperatives have been installed in Moroccan cities (Guelmim, Sidi Ifni, Ait Baha, Rhamna, Al-Hoceima) for the packaging of fresh fruits and their transformation into products intended for human and animal consumption. Various products are made at these units, including cactus jam, canned cladodes fillets, dried flowers, cosmetics, and oil extracted from the seeds (Arba, 2009). These products will serve an important socio-economic role for both farmers and rural populations, and help to achieve sustainable development in rural areas. Cactus is, therefore, an adequate species for sustainable agriculture in Morocco.

In order to contribute to the valorization of Moroccan cactus seed oil, this study showed results concerning the influence of the origin of cactus seeds collected from six main planting sites in Morocco (Hoceima, Bejâad, Rhamna, Ait Baha, Tiznit and Sidi Ifni), on the physicochemical characteristics and chemical composition of cactus seeds and seed oil. In addition, accelerated Rancimat oxidation at 120 °C was used to rapidly assess the effect of geographic origin on the oxidative stability of the cactus seed oil. The results of this study may contribute to developing a national standard for cactus seed oil.

## 2 Materials and methods

### 2.1 Plant material, solvents, and reagents

**Fig. 1:**
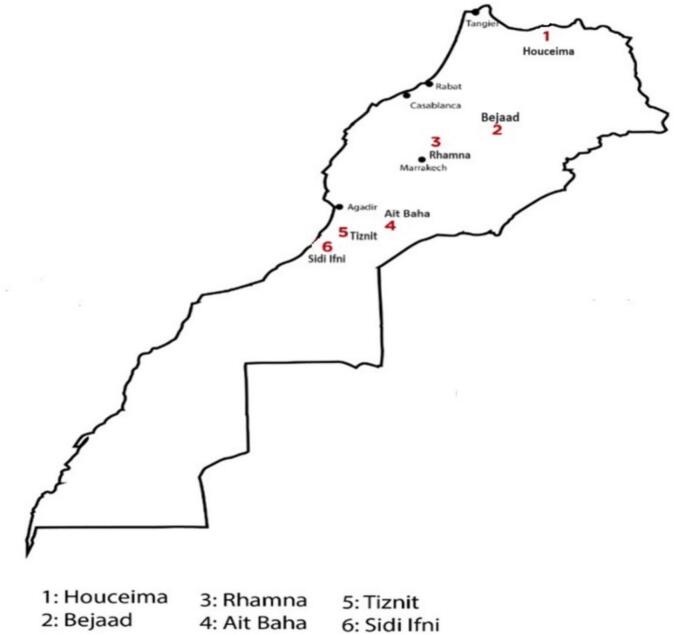
Location of the evaluated cactus seed sites of production.

Cactus seeds were collected in June 2017 from six cooperatives producing cactus seed oil located (Table 1, Fig. 1): Bejaad (*32°46′15” N, 6°23′28” W*), Ait Baha (30°4′7.9” N, 9°9′10” W), Rhamna (*32°28′12” N, 7°57′29” W*), Tiznit (29°42′1” N, 9°43′43” W), Hoceima (*35°14′41” N, 3°55′60” W*), and Sidi Ifni (*29°22′45” N, 10°10′17.6” W*).

**Table 1:** Geographical data of cactus seed collection sites.

	**Hoceima**	**Bejaâd**	**Rhamna**	**Ait Baha**	**Tiznit**	**Sidi Ifni**
**Altitude (meters above sea level)**	133	680	491	604	252	50
**Average temperature (°C)**	18.1	17.3	17.6	18.1	19.2	19.2
**Rainfall (mm/Year)**	272	438	312	229	158	133

The extraction was performed by using a KOMET D85 type worm press. The oils were then filtered and stored in brown glass bottles at 4 °C until analysis.

All reagents and solvents used were of analytical grade, except for the mobile phase used for HPLC, which was of chromatographic grade and purchased from VWR international (Darmstadt, Germany).

### 2.2 Moisture content, specific extinction, peroxide index (PV) and free fatty acids

All moisture content, free fatty acid, peroxide index (PV), and UV spectroscopy (Specific UV absorbance at 232 nm and 270 nm) were determined following the ISO 662:2016, DGF C—V 2 (06), DGF C-VI 6a Part 1 (05) and DGF C-IV 6 (13) (Dgf, 1998) methods, respectively.

### 2.3 Protein content

The nitrogen content was determined using the Kjeldahl procedure with Gerhardt model Vapodest 20 instrument. A factor of 6.25 was then used to convert the measured nitrogen to protein content expressed as a percentage (g/100 g) (Deutsche Gesellschaft für Fettwissenschaften).

### 2.4 Triacylglycerol composition

The DGF C-VI 14 (08) (Dgf, 1998) method was used to determine the triglyceride composition. The analysis was carried out with an Agilent 6890 gas chromatography system combined with an Agilent 7683B injector (Waldbronn, Germany) and equipped with a flame ionization detector (FID) and a RTX-65 column (30 m × 0.32 mm i.d, 0.1 μm film thickness). The injected volume was 1 μL of a solution of oil in isooctane (0.5 mg/mL). Carrier gas used was hydrogen (flow rate 1 mL/min). The oven temperature was programmed from 300 to 360 (2 °C/min). Injector and detector temperature were set at 380 °C. Triacylglycerols were identified by comparing their retention time, under the same analytical conditions, to that of other oils and data from literature.

### 2.5 Fatty acid composition

Fatty acids were converted to fatty acid methyl esters (FAMEs), according to DGF C-VI 10a (00) (Dgf, 1998). FAMEs analysis was carried out with an Agilent HP5890 gas chromatography system (Waldbronn, Germany) equipped with a FID and a CP-Sil 88 capillary column (100 m × 250 μm i.d, 0.2 μm film thickness). The hydrogen was used as carrier gas (flow rate: 1 mL/min). Detector and injector temperature was 250 °C. The initial oven temperature of 155 °C was increased to 230 °C on a scale of 1.5 °C/min. The injection volume was 1 μL in a split mode (1:50). The standard mixture of fatty acid methyl esters (Sigma Chemical Co.) was used for the identification of the peaks.

### 2.6 Phytosterol composition

Phytosterol composition and content were measured according to the DGF F-III 1 (98) method (Dgf, 1998). After a trimethylsilylation of the crude sterol fraction, the composition was carried out using an Agilent 6890 gas chromatography system equipped with a flame ionization detector. The SE 54 CB column capillary column (50 m × 320 μm i.d., 0.25 μm film thickness) was used. The carrier gas used was hydrogen with a flow rate of 1.6 mL/min. The oven temperature was programmed from 245 to 260 °C (5 °C/min). Injector and detector temperature was 320 °C. The injection volume was 1 μL in the split mode (1:20). The results were expressed as relative percentage of the area of each individual sterol peak to the area of all sterol peaks. Cholestan-3-ol was used as an internal standard.

### 2.7 Tocopherol composition

Tocopherol composition was determined according to the Method DGF F-II 4a (00) (Dgf, 1998). The analysis was conducted using a Merck-Hitachi low-pressure gradient system, equipped with a L-6000 pump, a fluorescence spectrophotometer (Merck-Hitachi F-1000) and a ChemStation integration system. Twenty μL of a filtrated solution (150 mg/mL of oil in n-heptane) was directly injected onto a Diol phase HPLC column (25 cm × 4.6 mm i.d.). A mixuture of *n*-heptane and tert-butyl methyl ether (99:1, V:V) was used as mobile phase, with a flow rate of 1.3 mL/min. Detector wavelengths were 295 nm for excitation, and 330 nm for emission. Identification was done using α-, β, δ-, and γ-tocopherol reference standards (chromatographic purity 97.6–99.6%, Merck KGaA, Darmstadt, Germany) and quantified through external calibration.

### 2.8 Oxidative stability

The oxidative stability of the extracted oil (3 g) was evaluated by the Rancimat method using a 743 Rancimat (Methrom AG, Herisau, Switzerland). The heating block was set at 120 °C with an air flow of 20 L/h.

### 2.9 Total phenol content and antioxidant activity

The total content of phenolic compounds extracted was determined using Folin-Ciocalteu reagent (Bouzid et al., 2023; Yoo, Lee, Park, Lee, & Hwang, 2004)). Briefly, 2.5 mL of diluted Folin-Ciocalteu reagent in water (1:10) and 4 mL of Na_2_CO_3_ (7.5%, *w*/*v*) were added to 0.5 mL of sample solution. The mixture was then allowed to stand at 45 °C in a water bath for 30 min and the absorbance measured at 765 nm using a UV–Vis spectrophotometer against a blank sample.

The antioxidant activity of cactus seed extracts was evaluated according to the method described by Scherer et al. (2009) (Scherer & Godoy, 2009). Briefly, 2.5 mL of plant extract was mixed with 0.5 mL of a 0.2 mM solution of DPPH (1.1-diphenyl-2-picrylhydrazyl) in ethanol. The mixuture was allowed to stand at room temperature for 30 min and the absorbance was measured at 517 nm using a UV–Vis spectrophotometer against blank samples.

### 2.10 Data analysis

Data are presented as means ± standard deviation and analysis of variance was performed with Tukey's test at (95% confidence level) using the software IBM SPSS Statistics 21. Grouped barplot representing the influence of location on oil quality parameters was illustrated using RStudio. Associations between physicochemical parameters of cactus seed oils in this study were performed using the Pearson correlation coefficient (r) with the metan package using RStudio version 1.3.1093.

## 3 Results and discussion

### 3.1 Seed analysis

#### 3.1.1 Moisture content

Water content in oil-containing seeds should be monitored with precaution to avoid oxidation risk. The results in Table 2 showed that the moisture content of seeds from different provenances ranges from 4.25 ± 0.1 to 8.97 ± 0.3 g/100 g. The average is around 8% and agrees with the values proposed for the safe storage of oil seeds (Bouzid et al., 2023; Yoo et al., 2004). Our results corroborate those reported in the literature (De Wit, Hugo, & Shongwe, 2017; El Mannoubi, Barrek, Skanji, Casabianca, & Zarrouk, 2009). Furthermore, the results showed an effect of the origin of the seeds on the moisture content, with zones of a high altitude (Ait Baha and Bejaâd) having a lower moisture content than the zones of low altitude (Hoceima, Rhamna, Tiznit and Sidi Ifni). Indeed, cactus seeds are generally dried in the open air under sunshine. Farmers judge the dryness of the seeds according to their knowledge before storing them in different polyethylene bags. Therefore, the drying process and storage facilities could explain the recorded moisture values.

**Table 2:** Effect of seeds geographical origin on moisture content, total protein, oil yield, total polyphenols content (TPC) and antioxidant power.

	**Hoceima**	**Bejaâd**	**Rhamna**	**Ait Baha**	**Tiznit**	**Sidi Ifni**
**Moisture (%)**	8.05 ± 0.2^a^	4.25 ± 0.1^b^	8.4 ± 0.3^a^	7.0 ± 0.1^c^	8.97 ± 0.3^d^	8.05 ± 0.3^a^
**Total proteins (%)**	8.25 ± 0.08^a^	7.98 ± 0.27^ab^	7.78 ± 0.19^bc^	7.5 ± 0.02^c^	8.24 ± 0.29^a^	7.01 ± 0.2^d^
**Oil yield (%)**	4.4 ± 0.5^a^	4.2 ± 0.1^a^	3.4 ± 0.2^b^	2.7 ± 0.1^c^	5.0 ± 0.4^d^	4.1 ± 0.2^a^
**TPC (mg EAG/100 g)**	165.6 ± 1.1^a^	187.0 ± 1.5^b^	208.5 ± 1.9^c^	220.7 ± 1.7^d^	215.5 ± 1.8^e^	225.9 ± 2.1^f^
**IC**_**50**_**value (mg/mL)**	1.03 ± 0.05^a^	0.77 ± 0.04^bc^	0.9 ± 0.03^ab^	0.72 ± 0.3^bc^	0.53 ± 0.06^c^	0.58 ± 0.1^c^

Mean values ± SD of determination for triplicate samples. Means followed by similar letters superscript in the same line are not significantly different according to the Tukey's test (p < 0.05)*.*

#### 3.1.2 Total proteins

The cactus fruit is an important source of biomolecules like proteins (Taoufik et al., 2015). The protein content of cactus seeds according to the geographical origin is presented in Table 2. Our results showed that the seeds protein content varied from 7.01 to 8.25 g/100 g of dry matter. These values were similar to those described by Tlili et al. (2011) (Tlili et al., 2011), but lower then those obtained from Tunisian cactus seeds (17.34 g/100 g) (Albergamo et al., 2022). Geographical origin had a significant influence (*p* < 0.05) on the protein content. The highest protein contents were recorded for the seeds of Hoceima (8.25 g/100 g) and Tiznit (8.24 g/100 g), while Sidi Ifni seeds (7.01 g/100 g) showed the lowest content.

#### 3.1.3 Oil yield

The results in Table 2 show that the oil content varied between 2.7 g/100 g (Ait Baha) and 5.0 g/100 g (Tiznit), suggesting that the origin of the seeds influenced the oil yield. Similar oil yields were reported for 12 cold-pressed South African cactus seeds (2.51 to 5.96 g/100 g) (De Wit, Motsamai, & Hugo, 2021). In contrast, our results were different to those reported by Albergamo et al. (2022) from Tunisian cactus seeds (9.65 g/100 g) (Albergamo et al., 2022).

Different moisture levels (4 to 9 g/100 g) did not affect the oil content of cactus seeds. The same phenomenon was observed for grape seed extraction, where the authors showed that at low moisture levels (5.5 to 7.5 g/100 g) no effect on oil yield was observed (Rombaut et al., 2015). Several factors can influence the yield of cactus seed oil, such as soil and climatic conditions, environmental conditions, and extraction methods (Matthäus & Özcan, 2011; Sakar et al., 2022).

#### 3.1.4 Total phenols content and antioxidant activity

Our results showed that the total polyphenol content in the water/methanol (20/80) extracts varied from 165 to 225 mg EAG/100 g of extract. The highest content was found in Sidi Ifni seed extract (225.9 ± 0.05 mg EAG/100 g of extract), followed by the extracts from Ait Baha (220.7 ± 0.05 mg EAG/100 g), Tiznit (215.5 ± 1.8 mg EAG/100 g) and Rhamna (208.5 ± 0.04 mg EAG/100 g). However, the seed extracts from Bejâad and Hoceima were less rich in polyphenols (187.0 ± 0.03 and 165.6 ± 0.03 mg EAG/100 g, respectively).

Our results were similar to those of Tlili et al. (2011) for Tunisian cactus seeds (222 mg EAG/100 g of extracts) (Tlili et al., 2011)**.** Albano et al. (2015) worked on two cactus genotypes (purple and orange fruit) from Italy (Albano et al., 2015)**.** They reported a total polyphenol content of 89.2 mg EAG/100 g fresh weight for the purple fruit and 69.8 mg EAG/100 g for the orange fruit. Factors such as cultivar type, climatic conditions and soil composition could explain these variations (Bijla et al., 2021; Gagour et al., 2022; Ibourki et al., 2021; Ibourki et al., 2022). A deep study of the phenolic profile of Moroccan cactus seed oil was done by Chbani et al. (2020). The authors identified 7 polyphenol compounds present in Moroccan cactus seed oil with a predominance of vanillin (3.9 mg/kg – 32.4 mg/kg), syringaldehyde (2.3 mg/kg – 12.3 mg/kg), and ferulaldehyde (2.6 mg/kg – 5.7 mg/kg) in all the studied regions. They concluded that the quantitatve variations between oils from different origins were the result of different climatic, storage or different processing conditions (Chbani et al., 2020).

Statistical analysis of our results showed that the total polyphenol contents in seed extracts depend on the origin of the seeds. A negative correlation between TPC and Rainfall (mm) (r^2^ = −0.6002) was recorded. We noticed that the concentration of total polyphenols increased significantly from north to south. This can be explained by increasing aridity, inducing stress favouring the appearance of polyphenols.

Polyphenols have significant antioxidant power which was tested using the DPPH test. The results in Table 2 show that there is a significant difference between regions. Tiznit and Sidi Ifni seeds recorded the lowest IC_50_ value (around 0.5 mg/mL). However, the seeds from Hoceima region showed the lowest antioxidant activity (IC_50_ = 1.03 mg/mL).

Our results also showed that the IC_50_ is negatively correlated (r^2^ = −0.722) with the content of phenolic compounds. Indeed, the seeds from Sidi Ifni and Tiznit had significant antioxidant power and a high polyphenol content compared to other origins. Other studies also showed that anti-radical activity is correlated with the level of polyphenols and flavonoids in extracts from medicinal plants (Ait Bouzid et al., 2022; Bijla et al., 2021; Gagour et al., 2022; Ibourki et al., 2022)().

### 3.2 Oils analysis

#### 3.2.1 Free fatty acids

**Fig. 2:**
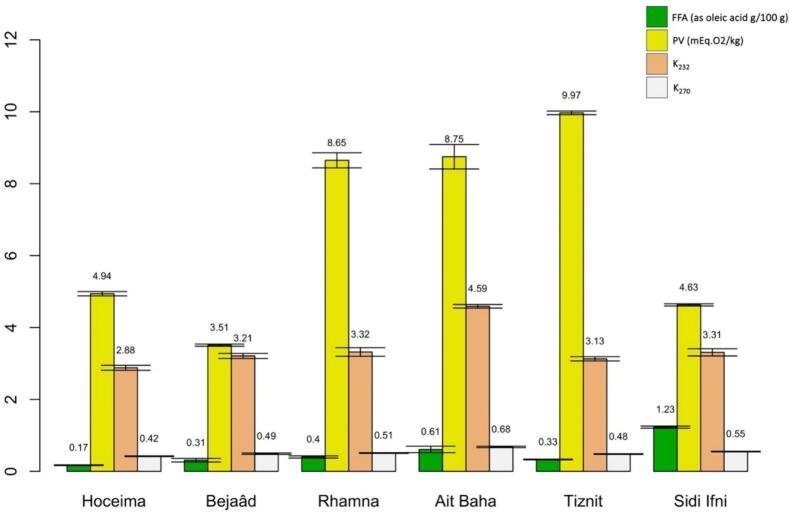
Effect of seeds geographical origin on acidity (FFA), peroxide value (PV) and specific extinctions (232 and 270 nm).

The acidity, referred to as free fatty acids (FFA), represents the proportion of free fatty acids produced by the hydrolysis of triacylglycerols in the oil. It is a parameter that indicates the quality of the vegetable oil (Gharby et al., 2012; Gharby & Charrouf, 2022). The results in Table S1 and Fig. 2 show that the FFA does not exceed 1.3% (as oleic acid %). Özcan and Al Juhaimi (2011) reported a similar value (1.41%) for Turkish prickly pear seed oil (Özcan & Al Juhaimi, 2011). Our results showed that different cactus seed oils studied (Hoceima, Bejaâd, Rhamna, Ait Baha, Tiznit and Sidi Ifni) respected the Codex Alimentarius, (2019) limit of 2 g of free oleic acid per 100 g of oil) for virgin oils. The very low level of acidity in all samples (<1.3%) indicates no enzymatic or chemical hydrolysis of triacylglycerols and demonstrates the excellent quality of the oils from different locations.

The geographical origin of the seeds had a significant influence (*p* < 0.05) on the FFA of the oil. The average acidity value was 0.5%, with the lowest value of 0.17% for Hoceima oil and the highest value of 1.23% for Sidi Ifni oil.

#### 3.2.2 Peroxide value (PV)

The obtained results showed that all samples had a peroxide value between 3 and 10 mEq.O_2_/kg (Table S1 and Fig. 2), which remains below the limit of 20 mEq.O_2_/kg according to the Codex Alimentarius Commission (2019). De Wit et al. (2017) obtained a higher peroxide value for South African cactus seed oils varying between 9.50 and 33.67 mEq.O_2_/kg **(**De Wit et al., 2017**).** Compared to other oils, the relatively high PVs of cactus seed oil can be explained by its high content of unsaturated fatty acids such as linoleic acid, which can lead to limited stability.

The statistical analysis of our results showed that the geographical origin had an influence (*p* < 0.05) on the peroxide value of our oils and an average positive correlation was found between the peroxide value and the moisture content (r^2^ = 0.604). High humidity promotes the formation of peroxides (Womeni et al., 2003). The regions of Tiznit, Ait Baha and Rhamna recorded the highest values from 8.65 to 9.97 mEq.O_2_/kg. The lowest value was obtained in oil from the region of Bejâad (3.51 mEq.O_2_/kg).

#### 3.2.3 Specific extinction (K_232_ & K_270_)

The analysis of specific absorbance showed that all the oils studied had K_232_ absorbance values ranging between 2.8 and 4.6 and specific K_270_ values between 0.4 and 0.7 (Table S1 and Fig. 2). Zine, Gharby, and El Hadek (2013) reported lower values for K_232_ (1.72) and K_270_ (0.31) (Zine et al., 2013).

**Fig. 3:**
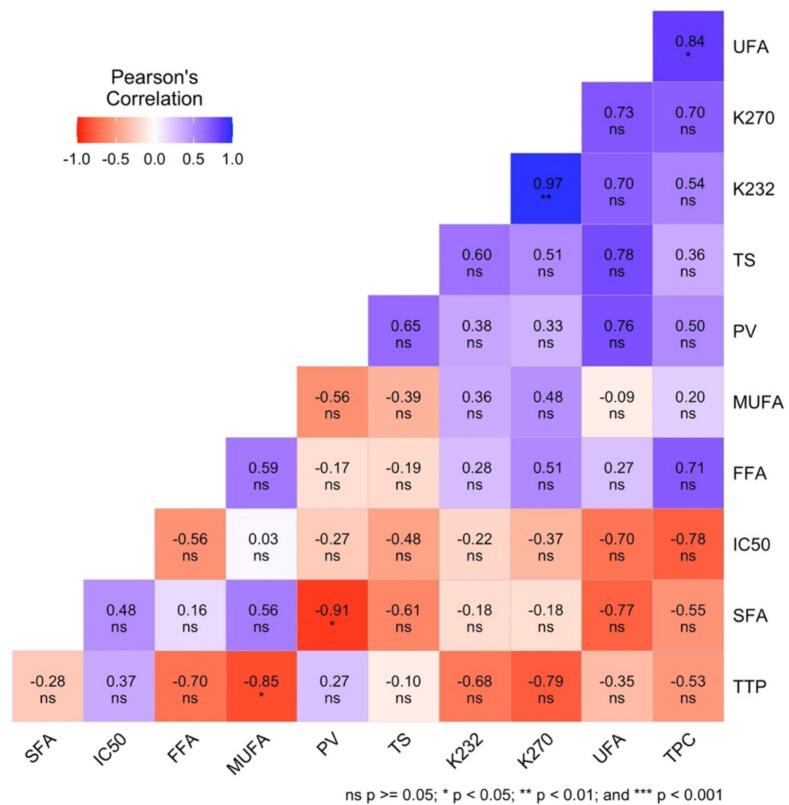
Pearson's correlation between the variables: FFA, PV, K_232_, K_270_, Total sterol (TS), Total tocopherol (TTP), SFA, UFA, Polyphenol, and DPPH (1/IC50 value) of the different samples of cactus oils.

The origin of the seeds influenced the specific coefficients K_232_ and K_270_. The highest values were recorded for oil from Ait Baha, while the lower values were observed for oil from Hoceima. The strong and positive correlation between K_232_ and K_270_ (r^2^ = 0.969, Table S2, Table S3 and Fig. 3) explains the rapid transformation of primary oxidation products into secondary products.

#### 3.2.4 Triacylglycerols composition

The glyceric fraction of cactus seed oil is mainly composed of triacylglycerols (93.37%). Diacylglycerols and monoacylglycerols represent only 2.6 and 0.2%, respectively. Free fatty acids were determined at 1.6%. Eleven triacylglycerols have been identified (Table 3). The three major compounds were palmito-dilinoleic (PLL) (19.9–24.3%), oleo-dilinoleic (LLO) (17.8–19.12%) and trilinoleic (LLL) (17.3–21.28%). Minor triacylglycerols were trioleic (OOO) (0.6–1.6%), palmito-diolein (POO) (1.3–2.7%), and oleo-dipalmitic (POP) (0.8–1.3%). Comparable results were found for the Tunisian cactus seed oil with a predominance of LLL (25%) OLL (21%), and PLL (15%) (El Mannoubi et al., 2009). The results in Table 3 show that the geographic origin of cactus seed oil had little influence on the triacylglycerols composition.

**Table 3:** Effect of seeds origin on the triglyceride composition of cactus seed oil (%).

	**POP**	**PLP**	**POO**	**PLS**	**PLO**	**PLL**	**OOO**	**SLO**	**OLO**	**LLO**	**LLL**
**Hoceima**	1.1 ± 0.1^a^	5.5 ± 0.2^a^	1.9 ± 0.1^ab^	3.2 ± 0.5^a^	10.1 ± 1.1^ab^	24.3 ± 2.7^a^	0.6 ± 0.1^a^	2.4 ± 0.2^a^	3.8 ± 0.5^a^	17.9 ± 1.1^a^	19.6 ± 1.9^ab^
**Bejaâd**	1.2 ± 0.2^a^	4.4 ± 0.3^bd^	2.3 ± 0.8^b^	3.3 ± 0.4^a^	10.4 ± 1.3^ab^	20.9 ± 0.8^ab^	1.6 ± 0.2^b^	3.6 ± 0.9^b^	5.0 ± 1.1^ab^	18.3 ± 1.6^a^	17.3 ± 2.3^a^
**Rhamna**	1.1 ± 0.5^a^	5.0 ± 0.1^abc^	2.1 ± 0.2^ab^	3.0 ± 0.8^a^	10.4 ± 1.5^ab^	22.5 ± 1.8^ab^	0.8 ± 0.1^a^	2.8 ± 0.7^ab^	4.6 ± 0.9^ab^	18.0 ± 2.6^a^	18.2 ± 0.9^a^
**Ait Baha**	1.1 ± 0.3^a^	4.2 ± 0.5^d^	2.1 ± 0.3^ab^	2.9 ± 0.6^a^	9.9 ± 0.9^ab^	19.9 ± 2.1^b^	1.1 ± 0.8^ab^	3.8 ± 0.9^b^	5.2 ± 1.2^ab^	19.1 ± 1.8^a^	18.4 ± 1.4^a^
**Tiznit**	0.8 ± 0.1^a^	5.2 ± 0.8^ac^	1.3 ± 0.1^a^	2.5 ± 0.1^a^	8.7 ± 1.1^a^	22.8 ± 2.5^ab^	0.8 ± 0.1^a^	2.8 ± 0.3^ab^	3.7 ± 0.5a	17.8 ± 1.5^a^	21.8 ± 2.4^b^
**Sidi Ifni**	1.3 ± 0.6^a^	4.6 ± 0.2^bcd^	2.7 ± 0.7^b^	3.2 ± 0.5^a^	11.4 ± 0.8^b^	20.8 ± 1.9^ab^	1.1 ± 0.3^ab^	3.4 ± 0.4^ab^	5.6 ± 0.7^b^	18.7 ± 3.1^a^	16.6 ± 1.4^a^

Mean values ± SD of determination for triplicate samples. Means followed by similar letters superscript in the same line are not significantly different according to the Tukey's test (p < 0.05). O, oleic acid; L, linoleic acid; P, palmitic acid; S, stearic acid.

#### 3.2.5 Fatty acids composition

The fatty acid composition of different cactus seed oils was determined after the trans-esterification of fatty acids to methyl esters. The results obtained are reported in Table 4.

**Table 4:** Effect of seeds origin on fatty acids composition (%) of cactus seed oil.

**Fatty acid**		**Hoceima**	**Bejaâd**	**Rhamna**	**Ait Baha**	**Tiznit**	**Sidi Ifni**
**Palmitic acid**	C16:0	11.4 ± 0.09^a^	10.7 ± 0.05^b^	11.1 ± 0.03^c^	10.18 ± 0.08^d^	11.4 ± 0.04^a^	10.9 ± 0.04^e^
**Palmitoleic acid**	C16:1	0.6 ± 0.01^a^	0.6 ± 0.01^a^	0.6 ± 0.01^a^	0.6 ± 0.01^a^	0.5 ± 0.01^b^	0.6 ± 0.01^a^
**Stearic acid**	C18:0	3.6 ± 0.01^a^	4.1 ± 0.01^b^	3.4 ± 0.008^c^	3.7 ± 0.01^d^	3.1 ± 0.01^e^	3.8 ± 0.01^f^
**Oleic acid**	C18:1Δ9	14.8 ± 0.1^a^	17.6 ± 0.04^b^	16.5 ± 0.02^c^	17.3 ± 0.01^b^	13.5 ± 0.03^d^	18.7 ± 0.02^e^
**Elaidic acid**	C18:1Δ9	0.3 ± 0.01^a^	0.3 ± 0.01^a^	0.3 ± 0.01^a^	0.2 ± 0.01^b^	0.1 ± 0.01^c^	0.2 ± 0.01^b^
**Vaccenic acid**	C18:1Δ11	4.9 ± 0.03^a^	5.2 ± 0.03^b^	5.0 ± 0.01^c^	4.9 ± 0.01^a^	4.3 ± 0.01^d^	4.8 ± 0.02^e^
**Linoleic acid**	C18:2	59.8 ± 1.2^a^	57.3 ± 0.5^a^	59.1 ± 2.1^a^	59.2 ± 1.2^a^	63.8 ± 3.3^b^	57.1 ± 1.1^a^
**Linolenic acid**	C18:3	0.2 ± 0.01^a^	0.2 ± 0.02^a^	0.2 ± 0.01^a^	0.3 ± 0.03^a^	0.2 ± 0.01^a^	0.2 ± 0.01^a^
**Arachidic acid**	C20:0	0.4 ± 0.02^a^	0.4 ± 0.01^a^	0.4 ± 0.03^a^	0.4 ± 0.01^a^	0.4 ± 0.02^a^	0.4 ± 0.01^a^
**Gadoleic acid**	C20:1	0.2 ± 0.01^a^	0.2 ± 0.02^a^	0.3 ± 0.03^a^	0.3 ± 0.01^a^	0.2 ± 0.06^a^	0.3 ± 0.05^a^
**Behenic acid**	C22:0	0.2 ± 0.04^a^	0.2 ± 0.02^a^	0.2 ± 0.01^a^	0.2 ± 0.03^a^	0.2 ± 0.01^a^	0.2 ± 0.05^a^
**SFA**	–	15.8 ± 2.3	15.7 ± 1.9	15.4 ± 0.5	15.5 ± 1.6	15.2 ± 0.4	15.7 ± 0.9
**UFA**	–	81.4 ± 3.5	82.1 ± 1.6	82.5 ± 0.9	83.3 ± 1.3	83.1 ± 1.1	82.4 ± 0.8
**MUFA**	–	21.0 ± 1.8	24.2 ± 0.4	22.8 ± 1.3	23.5 ± 0.5	18.8 ± 2.1	24.7 ± 0.3

Mean values ± SD of determination for triplicate samples. Means followed by similar letters superscript in the same line are not significantly different according to the Tukey's test (p < 0.05). SFA-saturated fatty acids, UFA-unsaturated fatty acids, MUFA monounsaturated fatty acids.

The cactus seed oil contains >81% unsaturated fatty acids, regardless of their geographical origin. The main unsaturated fatty acids were linoleic acid (57.1 to 63.8%) and oleic acid (13.5 to 18.7%). Therefore, cactus seed oil belongs to the group of oleic-linoleic oils. The minor unsaturated fatty acids were palmitoleic acid (0.6%), linolenic acid and gadoleic acid at a content of 0.2%. The cactus seed oil also contained 15% saturated fatty acids, mainly palmitic acid (10.1 to 11.4%) and stearic acid (3.1 to 4.1%). The fatty acid contents obtained in this study were similar to those described by Matthäus and Özcan (2011) (Matthäus & Özcan, 2011), who reported a predominance of linoleic acid (49.3 (Kepez) to 62.1% (Hatay-2)), oleic acid (13.0 (Hatay-2) to 23.5% (Kepez)) and palmitic acid (10.6 (Mut) to 12.8% (Kepez)). In another study, Nkoi, Wit, Fouche, Coetzer, and Hugo (2021) found that higher nitrogen fertilization rates significantly increased the oleic and stearic acid content. In contrast, the content of palmitic acid and *cis*-vaccenic acid decreased. However, linoleic fatty acid was not significantly affected (Nkoi et al., 2021). The richness of cactus seed oil in UFA leads to forming the main oxidation products. This finding tends to confirm the previous results with the PV (r^2^ = 0.816), K_232_ (r^2^ = 0.676) and K_270_ (r^2^ = 0.714) indices.

A negative correlation (r^2^ = −0.834, Table S2 and Fig. 3) was noted between SFA and UFA. An increase in UFA caused a decrease in SFA, which disturbs the stability of the oil due to the increase in double bonds. A negative correlation between SFA and the peroxide value was therefore expected.

The fatty acids in our oils were not significantly different, depending on the geographical origin. This indicates that localization only slightly influenced the composition of fatty acids. These results differ from those reported by Matthäus and Özcan (2011) (Matthäus & Özcan, 2011). They showed that the fatty acid composition of cactus seeds grown in different locations in Turkey differed greatly.

#### 3.2.6 Phytosterols composition

The total phytosterol content of the unsaponifiable fraction of cactus seed oil from different sources ranged between 8000 and 11,100 mg/kg (Table 5). The geographic origin of the seeds significantly influenced the amount of total phytosterols. The highest levels were observed for cactus seed oils from Ait Baha (11,091 mg/kg) and Tiznit (10,859 mg/kg), which were significantly different from a second cluster that includes Hoceima (8547 mg/kg), Rhaman (8492 mg/kg).

**Table 5:** Effect of seeds origin on individual and total sterol composition of cactus seed oil.

**Phytosterols (mg/kg)**	**Hoceima**	**Bejaâd**	**Rhamna**	**Ait Baha**	**Tiznit**	**Sidi Ifni**
**Total sterols**	8547 ± 199^a^	9207 ± 154^b^	8492 ± 308^a^	11,091 ± 327^c^	10,859 ± 306^c^	8292 ± 11^a^
**Campesterol**	837.6 ± 0.7^a^	892.1 ± 1.6^a^	811.8 ± 0.9^a^	1405.2 ± 1.4^b^	1395.4 ± 0.4^b^	839.1 ± 0.1^a^
**Stigmasterol**	119.6 ± 0.1^a^	165.7 ± 0.1^bc^	131.6 ± 0.1^ab^	222.9 ± 0.2^c^	223.7 ± 0.1^c^	145.9 ± 0.4^abc^
**Δ-7-Campesterol**	135.9 ± 0.2^a^	167.7 ± 0.^2a^	138.4 ± 0.1^a^	226.2 ± 0.3^a^	230.2 ± 0.6^a^	136.8 ± 0.2^a^
**Δ − 5,23-stigmastadienol**	99.1 ± 0.4^a^	116.9 ± 0.1^a^	106.1 ± 0.3^a^	133.1 ± 0.1^a^	152.0 ± 0.2^a^	97.0 ± 0.5^a^
**β-Sitosterol**	6151.3 ± 1.9^a^	6490.9 ± 2.1^ab^	6111.7 ± 2.5^a^	7142.6 ± 3.1^c^	7114.8 ± 4^bc^	5973.5 ± 2.9^a^
**Sitostanol**	275.2 ± 0.3^a^	342.5 ± 0.9^a^	288.7 ± 0.7^a^	364.9 ± 0.8^a^	377.9 ± 0.5^a^	282.7 ± 1.2^a^
**Δ − 5-Avenasterol**	391.4 ± 1.2^a^	462.2 ± 1.2^a^	360.0 ± 1.9^a^	473.6 ± 1.1^a^	384.4 ± 1.3^a^	339.1 ± 0.2^a^
**Δ − 5,24-stigmastadienol**	98.3 ± 0.1^ab^	118.8 ± 0.2^b^	85.8 ± 0.1^ac^	99.8 ± 0.1^c^	55.4 ± 0.1^d^	82.1 ± 0.1^ac^
**Δ − 7-Stigmasterol**	100.0 ± 0.1^a^	95.7 ± 0.1^a^	120.6 ± 0.1^a^	401.5 ± 0.5^b^	393.1 ± 0.9^b^	97.0 ± 0.3^a^
**Δ − 7-Avenasterol**	163.2 ± 0.2^ab^	187.8 ± 0.8^ab^	181.7 ± 0.6^ab^	326.1 ± 0.9^b^	255.2 ± 0.4a^b^	149.2 ± 0.2^a^

Mean values ± SD of determination for triplicate samples. Means followed by similar letters superscript in the same line are not significantly different according to the Tukey's test (p < 0.05).

The sterol fraction of cactus seed oil was mainly composed of β-sitosterol (5973–7142 mg/kg of total phytosterols). Other phytosterols were also detected; campesterol (9–13%), Δ-5-avenasterol (approximately 5%) and sitostanol (3%). The minor phytosterols were Δ-7-avenasterol, Δ-7-Stigmasterol, Δ-5,23-stigmastadienol, Δ-5,24-stigmastadienol and stigmasterol; their proportions did not exceed 4%.

#### 3.2.7 Tocopherols composition

The total tocopherol content of cactus seed oil ranged from 500 to 688 mg/kg (Table 6). The geographical origin of the seeds had a significant influence on the composition of tocopherols (*p* < 0.05). Cactus seed oil from Hoceima recorded the highest tocopherol content (687.3 mg/kg), followed by Tiznit oil (679.7 mg/kg), while Ait Baha oil showed the lowest content (502.1 mg/kg).

**Table 6:** Effect of seeds origin on individual, total tocopherol composition and oxidative induction time (OIT) of cactus seed oil.

**Tocopherols (mg/kg)**	**Hoceima**	**Bejaâd**	**Rhamna**	**Ait Baha**	**Tiznit**	**Sidi Ifni**
**Total tocopherols**	687.8 ± 3^a^	553.8 ± 2^b^	634.5 ± 2.5^c^	502.1 ± 2^d^	679.7 ± 4^e^	512.8 ± 3^f^
**α-Tocopherol**	9.9 ± 0.3^a^	23.0 ± 0.5^b^	18.3 ± 0.4^c^	30.4 ± 0.5^d^	10.4 ± 0.3^a^	17.6 ± 0.5^c^
**γ-Tocopherol**	654.5 ± 3^a^	496.0 ± 2^b^	596.7 ± 2.5^c^	445.3 ± 3.1^d^	649.8 ± 4^a^	473.7 ± 2.5^e^
**δ-Tocopherol**	10.7 ± 0.9^a^	9.0 ± 0.2^b^	5.4 ± 0.3^c^	5.9 ± 0.3^c^	7.3 ± 0.6^d^	5.9 ± 0.9^c^
**OIT (hours at 120** **°C)**	3.9 ± 0.4^ab^	3.5 ± 0.3^b^	3.0 ± 0.1^a^	2.2 ± 0.1^c^	3.1 ± 0.1^ab^	3.1 ± 0.2^ab^

Mean values ± SD of determination for triplicate samples. Means followed by similar letters superscript in the same line are not significantly different according to the Tukey's test (p < 0.05).

The major tocopherol was γ-tocopherol, representing on average 92% (445–654 mg/kg) of total tocopherols, followed by α-tocopherol (10–30 mg/kg) and δ-tocopherol (5.4–10.7 mg/kg), while β-tocopherol was not detected. These results agreed with the conclusions of other authors (Matthäus & Özcan, 2011; Ramadan & Mörsel, 2003). Furthermore, the origin of the seeds influenced individual tocopherols. Hoceima and Tiznit contained the highest level of γ-tocopherol (654.5 mg/kg and 649.8 mg/kg, respectively), while Ait Baha oil showed the lowest value (445.3 mg/kg). Studies have revealed a negative correlation between total tocopherol content and both altitude and distance from the coast in olive oil (Mohamed Mousa, Gerasopoulos, Metzidakis, & Kiritsakis, 1996) and argan oil (Elgadi et al., 2021). This phenomenon likely contributes to the elevated tocopherol levels observed in Hoceima. Moreover, water deficit conditions have been documented to enhance tocopherol levels (Carrera & Seguin, 2016). Nonetheless, the determination of significance is complicated by various confounding factors such as sunlight duration, relative humidity, and soil composition. Expanding the sample size may facilitate a more comprehensive understanding of the influence of these factors.

A significant positive correlation of α-tocopherol content with the increase in the level of K_270_ was observed (r^2^ = 0.853, Table S2, Table S3 and Fig. 3), which could be explained by its pro-oxidant activity in the first stages of the auto-oxidation.

#### 3.2.8 Oxidation induction time (OIT)

Oxidative stability is usually determined under standardized conditions, but accelerated methods can be used. Therefore, the Rancimat test was used for the present study to determine the induction period at 120 °C (393 K) (Table 6).

The oxidative stability of oils was slightly influenced by the geographic origin of the cactus seeds. The oil from Hoceima (3.5 h) and Bejâad (3.4 h) gave the highest OIT values compared to the oil from Ait Baha (2.2 h) at some conditions. The other origins (Rhamna, Tiznit and Sidi Ifni) recorded similar values of around 3.1 h. Nounah et al. (2021) reported that roasting the cactus seeds improved the oxidative stability of the oil. Induction time increased from 3.1 h for non-roasted seeds to 7.6 h after 40 min of roasting. In addition, the stability of cactus seed oil might be enhanced by mixing it with other oils (Nounah et al., 2021). Indeed, blending cactus seed oil with 25% Moringa oil is also a promising way to improve the stability of cactus seed oil (Salama et al., 2020). Gharby et al. (2021) and Taneva et al. (2021) have shown that cactus oil is too sensitive to oxidation compared to argan oil (Gharby et al., 2021; Taneva et al., 2021).

Regarding the sensitivity to oxidation of cactus seed oil due to its high linoleic acid content, special precautions, such as protection against heat and light, should be considered for the prolonged storage of seed cactus oil (Gharby et al., 2012; Gharby et al., 2018; Harhar et al., 2011; Harhar, Gharby, Guillaume, & Charrouf, 2010).

Significant negative correlations were reported between OIT and, PV (r^2^ = −0.6036), K_232_ (r^2^ = −0.9367) and K_270_ (r^2^ = −0.9163). An increase in these values resulted in a decrease in the OIT value. This correlation showed that the OIT values of these oils were highly dependent on the primary and secondary oxidation products (Gharby et al., 2011; Gharby et al., 2023; Hajib et al., 2021).

The low oxidative stability and the variability of OIT values between origins could be explained by the level of tocopherols, phytosterols and unsaturated fatty acids in cactus seed oil. Unsaturated fatty acids are very susceptible to oxidation reactions due to several double bonds; therefore, a negative correlation (r^2^ = −0.7818, Table S2, Table S3 and Fig. 3) between UFA and the OIT was observed. Table S2 reports a positive correlation of OIT with δ-tocopherol (r^2^ = 0.6492) and γ-tocopherol (r^2^ = 0.4902) suggesting an improved oxidative stability in the presence of tocopherols. The action of tocopherols on the oxidative stability of vegetable oils is a complex phenomenon because they are effective antioxidants at low concentrations. Still, they gradually lose their effectiveness as their concentrations in vegetable oils increase (Kamal-Eldin, 2006).

## 4 Conclusions

The present study shows that cactus seeds are rich in biomolecules (proteins, oil, and polyphenols) that may be appealing for the dermo-cosmetics industry. Indeed, the extracted polyphenols have strong antioxidant potential, suggesting an anti-ageing application. The study of the oil's physicochemical characteristics showed that the quality indices (acidity, peroxide value, and UV-absorbance) recorded slight variations depending on the geographical origin. These variations may be controlled if correct manufacturing practices, including packaging and storage, are followed. Cactus seed oil is an oleic-linoleic type, rich in phytosterols and tocopherols. Its high linoleic acid content suggests important nutritional and dermo-cosmetic properties; however, its oxidation resistance is low. The composition of triacylglycerols, fatty acids and phytosterols showed little variation depending on the geographical origin, while the origin might influence the tocopherol content. Cactus seed oil is the flagship product of the *O. ficus-indica* plant. Consequently, more efforts should be done to ensure its authenticity using quality markers such as phenolic or volatile compounds. Indeed, packaging and oil storage are critical steps in the production chain; therefore, optimization and storage experiments are recommended.

## CRediT authorship contribution statement

**Issmail Nounah:** Writing – original draft, Methodology. **Said El Harkaoui:** Writing – original draft, Methodology. **Ahmed Hajib:** Methodology, Data curation. **Said Gharby:** Validation, Conceptualization. **Hicham Harhar:** Supervision, Data curation. **Abdelhakim Bouyahya:** Writing – review & editing, Supervision. **Giovanni Caprioli:** Writing – review & editing, Supervision, Resources. **Filippo Maggi:** Supervision. **Bertrand Matthäus:** Supervision, Software, Resources. **Zoubida Charrouf:** Project administration.

## Declaration of competing interest

The authors declare that they have no known competing financial interests or personal relationships that could have appeared to influence the work reported in this paper.

## Data Availability

The authors are unable or have chosen not to specify which data has been used.

## Appendix A Supplementary data

Supplementary material Image 1
