# Supplementary material for: Effect of seed's geographical origin on cactus oil physico-chemical characteristics, oxidative stability, and antioxidant activity
Source: Food Chem X. 2024 May 7;22:101445. doi: 10.1016/j.fochx.2024.101445 (PMC11101876; doi:10.1016/j.fochx.2024.101445)
Supplement: Supplementary file 1 — Supplementary material [file mmc1.docx]

**Table S1:** Effect of seeds geographical origin on acidity (FFA), peroxide value (PV) and specific extinctions (232 and 270 nm).

|  |  | **Hoceima** | | **Bejaâd** | | **Rhamna** | | **Ait Baha** | | **Tiznit** | | **Sidi Ifni** |  |
| --- | --- | --- | --- | --- | --- | --- | --- | --- | --- | --- | --- | --- | --- |
| **FFA (as oleic acid g/100 g)** | | 0.17± 0.01^a^ | 0.31± 0.05^b^ | | 0.4± 0.03^c^ | | 0.61± 0.09^d^ | | 0.33± 0.01^bc^ | | 1.23 ± 0.03^e^ | | |
| **PV (mEq.O_2_/kg)** | | 4.94± 0.06^a^ | 3.51± 0.03^b^ | | 8.65± 0.21^c^ | | 8.75± 0.34^c^ | | 9.97± 0.05^d^ | | 4.63± 0.03^e^ | | |
| **K_232_** | | 2.88 ± 0.07^a^ | 3.21± 0.07^bc^ | | 3.32± 0.12^c^ | | 4.59± 0.05^d^ | | 3.13± 0.06^b^ | | 3.31 ± 0.1^c^ | | |
| **K_270_** | | 0.42± 0.01^a^ | 0.49± 0.02^bc^ | | 0.51± 0.01^c^ | | 0.68± 0.02^d^ | | 0.48± 0.01^b^ | | 0.55 ± 0.01^e^ | | |

*Mean values ± SD of determination for triplicate samples. Means followed by similar letters superscript in the same line are not significantly different according to the Tukey's test (p<0.05).*

**Table S2:** Pearson’s correlation matrix coefficient between the variables: FFA, PV, K_232_, K_270_, Total sterol, Total tocopherol, SFA, UFA, Polyphenol, and DPPH (1/IC_50_ value) of the different samples of cactus oils.

| Variables | FFA | PV | K232 | K270 | Total sterols | Total tocopherols | SFA | UFA | Polyphenols | DPPH  (1/IC_50_ value) |
| --- | --- | --- | --- | --- | --- | --- | --- | --- | --- | --- |
| FFA | **1** | -0.162 | 0.288 | 0.499 | -0.239 | -0.708 | -0.109 | 0.276 | 0.712 | 0.509 |
| PV |  | **1** | 0.386 | 0.345 | -0.201 | 0.270 | **-0.952** | **0.816** | 0.505 | 0.298 |
| K_232_ |  |  | **1** | **0.969** | **-0.936** | -0.676 | -0.419 | 0.676 | 0.537 | 0.084 |
| K_270_ |  |  |  | **1** | **-0.875** | -0.779 | -0.430 | 0.714 | 0.696 | 0.213 |
| Total sterols |  |  |  |  | **1** | 0.625 | 0.260 | -0.414 | -0.294 | 0.177 |
| Total tocopherols |  |  |  |  |  | **1** | -0.147 | -0.289 | -0.527 | -0.215 |
| SFA |  |  |  |  |  |  | **1** | **-0.834** | -0.649 | -0.390 |
| UFA |  |  |  |  |  |  |  | **1** | **0.851** | 0.604 |
| Polyphenols |  |  |  |  |  |  |  |  | **1** | 0.722 |
| DPPH (1/IC_50_ value) |  |  |  |  |  |  |  |  |  | **1** |

*The values in bold are different from 0 at a significance level alpha = 0.05.*

**Table S3:** *p*-values of the correlation matrix coefficient between all variables.

| Variables | FFA | PV | K_232_ | K_270_ | Total sterols | Total tocopherols | SFA | UFA | Polyphenols | DPPH  (1/IC_50_ value) |
| --- | --- | --- | --- | --- | --- | --- | --- | --- | --- | --- |
| FFA | **0** | 0.60 | 0.580 | 0.314 | 0.649 | 0.115 | 0.838 | 0.597 | 0.112 | 0.303 |
| PV |  | **0** | 0.450 | 0.503 | 0.702 | 0.605 | **0.003** | **0.048** | 0.307 | 0.566 |
| K_232_ |  |  | **0** | **0.001** | **0.006** | 0.141 | 0.408 | 0.140 | 0.271 | 0.875 |
| K_270_ |  |  |  | **0** | **0.022** | 0.068 | 0.394 | 0.111 | 0.125 | 0.686 |
| Total sterols |  |  |  |  | **0** | 0.184 | 0.619 | 0.415 | 0.571 | 0.737 |
| tocopherols |  |  |  |  |  | **0** | 0.781 | 0.579 | 0.282 | 0.682 |
| SFA |  |  |  |  |  |  | **0** | **0.039** | 0.163 | 0.445 |
| UFA |  |  |  |  |  |  |  | **0** | **0.032** | 0.205 |
| Polyphenols |  |  |  |  |  |  |  |  | **0** | 0.105 |
| DPPH(1/IC_50_ values) |  |  |  |  |  |  |  |  |  | **0** |
